# Research on distribution strategy of logistics enterprise alliance based on three-party evolution game

**DOI:** 10.1038/s41598-024-65723-9

**Published:** 2024-06-28

**Authors:** Wenxue Ran, Dandan He, Zhaoxia Li, Yun Xue, Zhenzhen He, Aravinda Dananjaya Basnayaka Basnayaka Gunarathnage

**Affiliations:** 1 School of Logistics and Management Engineering, Yunnan University of Finance and Economics, Kunming, 650221 China; 2 School of Accounting, Lanzhou University of Finance and Economics, Lanzhou, 730000 China

**Keywords:** Reward and punishment mechanism, Low-carbon preference, Collaborative distribution, Evolutionary game, Climate-change mitigation, Sustainability

## Abstract

Currently, collaborative distribution models have not reached the optimal state of carbon emissions. The cost of additional low-carbon expenditures and the problem of carbon data verification have led to the lack of motivation for reducing emissions among collaborative distribution enterprises. Therefore, how to incentivize them to adopt the low-carbon model is crucial for achieving low-carbon goal. By relying on a government-led digital platform, this paper designs a dual low-carbon incentive strategy to encourage enterprise-alliance to adopt a low-carbon distribution model. In this paper, we first construct an evolutionary game model of the government, enterprise-alliance and end-users; then we explore the conditions of the three-party equilibrium evolution strategy by solving the model and analyzing the stability; and finally, we conduct simulation validation and results analysis with the help of MATLAB. In summary, we found that government punishment is more effective at regulating enterprise-alliance than reward. End-users’ behavior is affected by the costs they need to bear, and they no longer support enterprise-alliance to carry out collaborative low-carbon distribution above a certain threshold.

## Introduction

In recent years, China's carbon emissions trading market has experienced unprecedented development, and the carbon trading mechanism has entered a new phase. At the same time, consumer attitudes have also changed, shifting from the original affordable to low-carbon environmental protection. Research shows that consumers' increased preference for low-carbon makes them pay more attention to the low-carbon attributes of products when they buy them, and they are willing to pay a high price for them. As a result, the end-users' low-carbon preference has become an important consideration in logistics distribution decisions^1^.

Logistics enterprise-alliance is a cooperative organization formed by a number of logistics enterprises to improve the overall efficiency and competitiveness of the logistics industry. Logistics enterprise alliance as a kind of enterprise relationship concluded voluntarily between enterprises. It has the characteristics of resource sharing, platform building and result sharing, so it can meet the individualized needs of logistics enterprises and make the relationship between logistics enterprises stable and long-term^2^. With the goal of "exploring the best opportunities for collective competitive advantage", the Alliance draws on the management philosophy of virtual enterprises and integrates them according to specific needs^3^. At present, there are enterprise alliances, such as cold chain logistics alliance, sea-railway logistics alliance, railroad logistics alliance, e-commerce logistics alliance, transportation logistics alliance, cross-border logistics alliance and so on.

**Figure 1:**
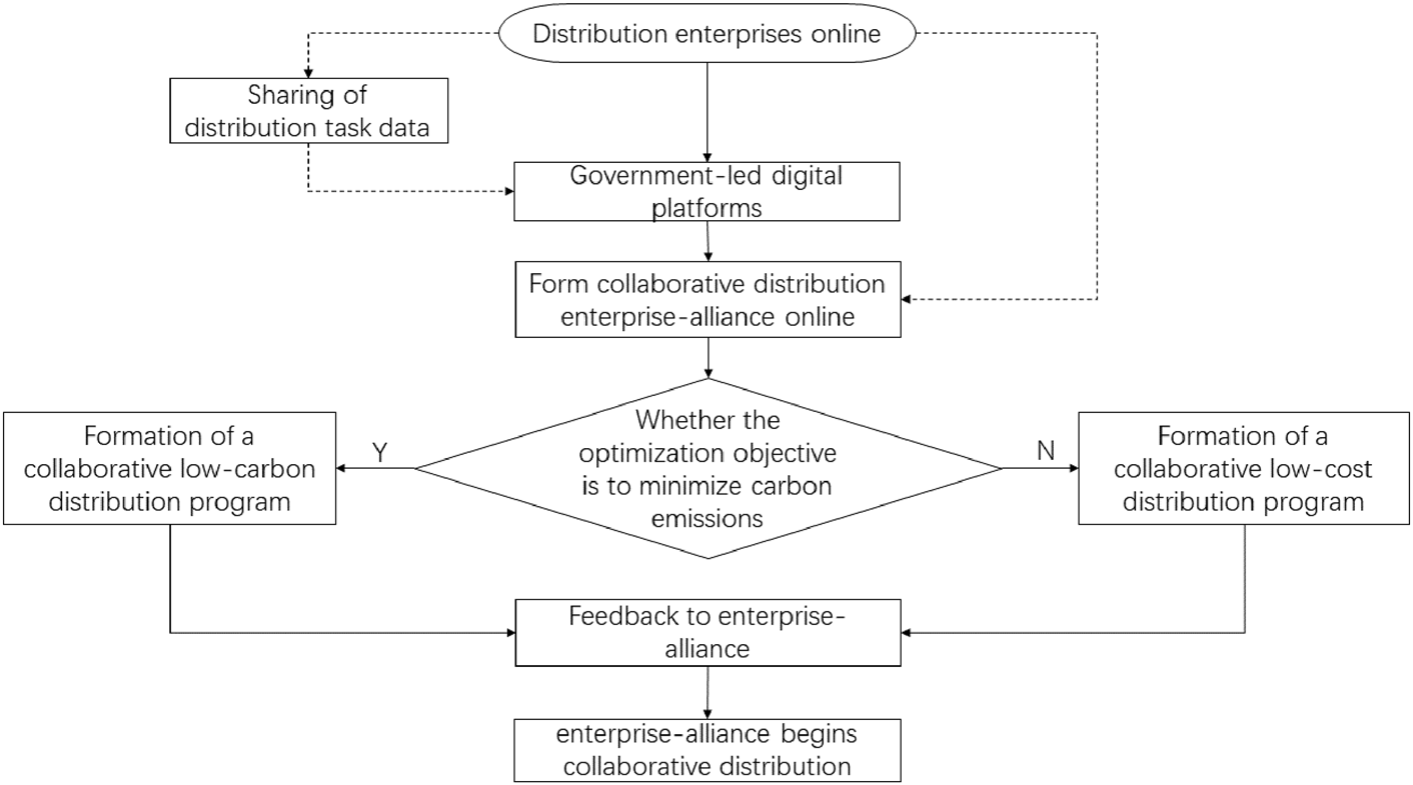
Schematic diagram of enterprise-alliance collaborative distribution.

Under the traditional model, logistics distribution enterprises have difficulties monitoring and managing carbon emissions, leading to inaccurate data and nontransparent supervision. To solve this problem, a collaborative distribution model with a digital platform has emerged^4^. Collaborative distribution refers to the modernized distribution method in which logistics enterprise alliance use online platforms to integrate distribution orders from different enterprises and jointly complete distribution tasks. In this way, distribution efficiency can be improved, and distribution costs and carbon emissions can be significantly reduced^5^. Collaborative distribution becomes more important with the boom of low-carbon goals and the in-depth study of low-carbon concepts. The digitization platform mentioned in this paper is led by the government and is currently free for distribution enterprises. In the initial stage, the government-run digital platform can incentivize distribution enterprises to participate in collaboration, account for carbon emissions in the distribution process, provide data support for low-carbon incentives, and effectively connect with the carbon trading market^6^ . The flowchart of the enterprise-alliance for collaborative distribution in this paper is shown in Fig. 1.

There are many studies on collaborative distribution among enterprises. Rao et al. designed an optimal price discount strategy to incentivize customers to participate in collaborative distribution^7^. Zhou et al. studied the optimization problem of collaborative distribution using vehicle piggybacking to carry out collaboration^8^. Qie et al. developed a cost sharing model based on the consensual function to solve the problem of cost allocation among enterprises^9^. Chu et al. argued that the joint distribution improves the delivery rate of express delivery in the last-mile logistics^10^. Rao et al. constructed a cost minimization collaborative distribution model to analyze the necessity of coalition splitting^11^. Improving vehicle utilization through a collaborative distribution approach enables firms to contribute to the three pillars of sustainable development by reducing fuel consumption and emissions, increasing profitability, and improving customer satisfaction^12^. Rao et al. proposed a vehicle make-up strategy of finding idle vehicles from within the alliance and then leasing vehicles from outside the alliance, and constructed a quantitative model for the cost of multi-party collaborative distribution considering the constraints on the distribution capacity of the enterprises^13^. Han et al. argued that the distribution of common profit has always been a key obstacle to the effective development of joint distribution in the context of green and low-carbon, and explored a fairer and more reasonable profit distribution scheme^14^. Chen et al. develop a cold chain logistics model considering joint distribution and carbon trading mechanism^15^; Rao et al. design a default recovery and loss compensation mechanism for members' withdrawal from an alliance in collaborative distribution^16^.

Evolutionary game theory is widely used to analyze the strategy selection problem under the participation of multiple subjects. Li et al. constructed a developer-government-consumer three-way evolutionary game model by combining the carbon trading prospect theory^17^. Based on evolutionary game theory, Zeng et al. explored the influence of platform reward and punishment mechanisms on the low-carbon strategy selection of manufacturing enterprises^18^. Lin et al. studied the strategic decision-making process of two participants in the context of medical malpractice based on the evolutionary game model of doctors and patients^19^. Wu et al. developed a game theory model involving packaging suppliers and logistics companies and based on the theory of corporate social responsibility^20^. Tang et al. constructed a three-party evolutionary game model to analyze the strategic choices of automobile companies, consumers and the Chinese government^21^. Gong et al. constructed a three-party evolutionary game model of cloud manufacturing platform, service provider and service demander, and analyzed the evolutionary stabilization strategy^22^. Yuan et al. constructed a three-party evolutionary game model to study the allocation of medical supplies in the rescue environment of public health emergencies under the condition of information incompleteness^23^. Wang et al. constructed an evolutionary game model of value co-creation in a cloud manufacturing innovation ecosystem, and studied the relationship between platform enterprises and government participation behaviors under dynamic and static reward and punishment mechanisms^24^. Qiu et al. constructed a three-party evolutionary game model of e-commerce platforms, merchants, and consumers, and examined the impacts of the influencing factors on the strategic choices of each party^25^. Chen et al. analyzed the game between the government and polluting enterprises under the environmental tax system through an evolutionary game model^26^.

From the above review, it can be seen that current researches on the collaborative distribution of logistics enterprise alliance have focused mainly on alliance splitting strategies, cost sharing methods and the mechanism of recovery and compensation for members' withdrawal from the alliance. Researches on collaborative distribution participants has considered only the influence of government regulatory decisions. In this paper, the distribution strategy of logistics enterprise alliance is investigated considering government reward and punishment as well as end-users' low-carbon preference. Therefore, this paper constructs an evolutionary game model of the government, enterprise-alliance and end-users, to consider the impact of the government's reward and punishment mechanism and end-users' low-carbon preference on the distribution strategy of enterprise-alliance. The research questions that are addressed in this paper are as follows: (1) From the analysis of the evolutionary route, what are the differences in the dynamic evolutionary process of the game subjects under different strategy choices of multiple subjects? (2) From the perspective of supply-side government policy analysis, what is the impact of government reward and punishment mechanism on the distribution strategy of logistics enterprise-alliance? (3) From the analysis of end-user behavior on the demand side, how does the end-user's low-carbon preference affect the distribution strategy of logistics enterprise-alliance?

## Model construction

### Description of the problem

**Figure 2:**
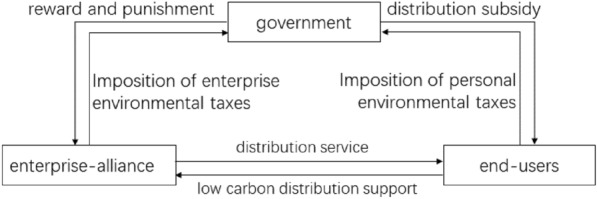
Logical relationship diagram of the evolution of the three-party evolutionary game.

The collaborative distribution of logistics enterprise-alliance can be categorized into collaborative low-cost distribution and collaborative low-carbon distribution according to the distribution mode^27^. The enterprise-alliance usually strives to maximize profits, so it prefers to choose collaborative low-cost distribution, while the government is committed to maximizing social benefits, and hopes that the enterprise-alliance will choose collaborative low-carbon distribution. Taxation is a kind of environmental regulation policy of the government, which can improve the environmental protection willingness of enterprise-alliance by levying environmental tax. At the same time, from the implementation level, the government needs to implement certain regulatory measures on the collaborative distribution strategy of enterprise-alliance. Reward for collaborative distribution enterprise-alliance that aim to minimize carbon emissions, and punishment for collaborative distribution enterprise-alliance that aim to minimize costs. The government, through taxation and reward and punishment mechanisms, encourages the alliance to optimize the distribution plan with the goal of carbon emissions, so as to achieve the goal of energy saving and emission reduction. Similarly, the government grants distribution subsidies to end-users with low carbon preferences, and imposes a personal environmental tax on end-users without low carbon preferences in order to optimize emissions reduction. Finally, end-users with low-carbon preferences are willing to pay additional low-carbon distribution subsidies to enterprise-alliance, which provide logistics and distribution services to end-users. Therefore, the mechanism of the three-way game between the government, enterprise-alliance and end-users in the distribution process is shown in the following Fig. 2.

### Model assumptions and parameter design

In assumption 1 for the natural environment, subject 1 for the government subgroup, subject 2 for the enterprise-alliance subgroup, and subject 3 for the end-user subgroup, assuming that the participating subjects have finite rationality, the model of the game parties is in the initial stage, the game strategy selection over time gradually evolves and stabilizes in the optimal strategy and the game process does not take into account the impact of other subjects on the game^28,29^.

Assumption 2 enterprise-alliance: the set of actions available to the enterprise-alliance is assumed to be{collaborative low-carbon distribution, collaborative low-cost distribution}^30^ . Collaborative low-cost distribution refers to an enterprise-alliance that minimizes costs as the goal of distribution route design, and collaborative low-carbon distribution refers to the enterprise-alliance that minimizes carbon emissions as the goal of distribution route design. Let the probability of collaborative low-carbon distribution of enterprise-alliance be $x$ ,the probability of collaborative low-cost distribution is $1-x$ where $0\le x\le 1$ .

Assumption 3 government: the set of actions available to the government is assumed to be{reward and punishment combination policy, flow in the form of policy}^31^ . The meaning of the policy of combining reward and punishment is that when the enterprise-alliance chooses low-carbon distribution or low-cost distribution, the government, as an external regulator, gives flexible reward or punishment to regulate the market, and the meaning of flow in the form of policy is that the government uses fixed rather than flexible reward or punishment to regulate the market. Let the probability that the government adopts the policy of combining reward and punishment be as follows $\text{y}$,the probability of adopting the flow in the form of policy is $1-\text{y}$ where $0\le \text{y}\le 1$.

Assumption 4 end-users: the set of actions available to the end-users is assumed to be{with low-carbon preference , without low-carbon preference} . End-users with low-carbon preference are willing to spend more money supporting collaborative low-carbon distribution through enterprise-alliance; End-users without low-carbon preference are more likely to support collaborative low-cost distribution through enterprise-alliance. Let the probability that end-users with low-carbon preference be $\text{z}$ , the probability of without low-carbon preference is $1-\text{z}$ where $0\le \text{z}\le 1$ . End-users who support low-carbon distribution have to pay higher distribution costs. To promote the development of low-carbon distribution, the government provides end-users with a certain amount of low-carbon distribution subsidies.

Assumption 5 in a scenario where end-users demand for goods remains constant but the unit price of distribution increases due to end-users' low-carbon preference, the benefits of collaborative distribution for the enterprise-alliance increase accordingly. When an enterprise-alliance chooses collaborative low-cost distribution or collaborative low-carbon distribution strategies, differences in optimized routes lead to differences in distribution costs. Therefore, different collaborative distribution strategies chosen by enterprises will affect the collaborative distribution costs, carbon emissions, and revenues of the enterprise-alliance. In addition, the distribution process includes the cost of cargo deployment in addition to the cost generated by the distribution itself^30^ .

Assumption 6 revenues^32^: revenues from collaborative distribution by the enterprise-alliance $\left({\varphi }_{1},{\varphi }_{2}\right)$, low-carbon distribution support $\beta {\mathcal{V}}$ that the enterprise-alliance receives from end-users; environmental taxes levied by the government on the enterprise-alliance $\left({\mathcalligra{m}}{\mathcal{E}}_{1},{\mathcalligra{m}}{\mathcal{E}}_{2}\right)$ and personal environmental taxes levied by the government on the end-users $\left(\gamma L,{D}_{2}\right)$, and penalties $A$ imposed by the government on enterprise-alliance for low-carbon distribution; end-users with low-carbon preference can receive the government's distribution subsidy $\left(\alpha {\mathcal{W}},{D}_{1}\right)$.

Assumption 7 costs^32^: the cost of collaborative distribution by the enterprise-alliance $\left({\mathcal{C}}_{x1},{\mathcal{C}}_{x2}\right)$, the deployment cost of collaborative distribution by the enterprise-alliance $\uptheta$; the regulatory costs invested by the government $\left({\mathcal{C}}_{y1},{\mathcal{C}}_{y2}\right)$, the government's bonus to the enterprise-alliance when it distributes low carbon $S$, and distribution subsidies for end-users with low-carbon preferences $\left(\alpha {\mathcal{W}},{D}_{1}\right)$; the end-users' costs are mainly the time cost $\updelta$ needed to support low-carbon distribution in terms of the low-carbon distribution support $\beta {\mathcal{V}}$ they are willing to pay to the enterprise-alliance.

Based on the above assumptions, the parameters and their significance required to comprehensively set up the three-party evolutionary game model are shown below:

Enterprise-alliance:

${\mathcal{C}}_{x1}$: Distribution costs of collaborative low-carbon distribution ${\mathcal{C}}_{x1}>0$

${\mathcal{C}}_{x2}$: Distribution costs for collaborative low-cost distribution ${\mathcal{C}}_{x2}>0$

$\uptheta$: Redeployment costs for collaborative distribution $\uptheta >0$

${\mathcal{E}}_{1}$: Carbon emissions when collaborating on low-carbon distribution ${\mathcal{E}}_{1}>0$

${\mathcal{E}}_{2}$: Carbon emissions when collaborating on low-cost distribution ${\mathcal{E}}_{2}>0$

${\varphi }_{1}$: Revenues of collaborative low-carbon distribution ${\varphi }_{1}>0$

${\varphi }_{2}$: Revenues of collaborative low-cost distribution ${\varphi }_{2}>0$

Governments:

${\mathcalligra{m}}$: Government's unit penalty factor for carbon emissions from corporate alliances (environmental tax rate^30^)$0\le {\mathcalligra{m}}\le 1$

$S$: Government reward for enterprise-alliance when collaborating on low-carbon distribution $S>0$

$A$: Government punishment when enterprise-alliance collaborate on low-cost distribution $A>0$

${\mathcal{C}}_{y1}$: Regulatory costs incurred by the government in adopting the policy of reward and punishment combination ${\mathcal{C}}_{y1}>0$

${\mathcal{C}}_{y2}:$ Regulatory costs incurred by governments in adopting the policy of flow in the form ${\mathcal{C}}_{y2}>0$

$\omega$: Environmental revenues to governments when enterprise-alliance collaborate on low-carbon distribution $\omega >0$

$\alpha{ \mathcal{W}}$: Distribution subsidies for end-users with low-carbon preference when the government adopts a policy of reward and punishment.${\mathcal{W}}$ is the cap of government reward

$\gamma L$: Individual environmental taxes on end-users without low-carbon preferences when the government adopts a policy of reward and punishment,^32^ $L$ is the upper limit of the government's tax revenue

${D}_{1}$: Distribution subsidies for end-users with low-carbon preference when the government adopt flow in the form policy

${D}_{2}$: Individual environmental taxes levied on end-users without low-carbon preferences when governments adopt flow in the form policy.

End-users:

$\beta {\mathcal{V}}$: End-users with low-carbon preference are willing to pay for low-carbon distribution support ${\mathcal{V}}$ is the upper limit of what end-users are willing to pay

$\updelta$: Cost of time for end-users to support low-carbon distribution.

### Game model construction and analysis

When logistics enterprise alliance choose distribution strategies, their behaviors are constrained by a variety of factors. To analyze the influencing factors of the behavior of each participating subject, evolutionary game theory can be used to construct an evolutionary game model^33^. First, according to the assumptions, the benefits of enterprise-alliance, end-users and government under different behavioral strategies are listed, and then their average expected benefits are calculated, and the replicated dynamic equations of each party are finally derived. The three parties, the enterprise-alliance, end-user and government obtain the benefits according to the benefit payment matrix in Tables 1 and 2 below , where the benefits of each subject is equal to the revenues of each subject minus its corresponding costs (the equations in rows 1–3 of each decision combination in the payment matrix represent the expected benefits of the government, the enterprise-alliance and the end-user under the decisions of the corresponding parties).

**Table 1:** Tri-party payment matrix under collaborative low-carbon distribution by enterprise-alliance $(x).$

Behavior of game subjects		End-users	
		With low-carbon preference	Without low-carbon preference
Government	Reward and punishment combination policy	${\mathcalligra{m}}{\mathcal{E}}_{1}+\omega -S-{\mathcal{C}}_{y1}-\alpha \mathcal{W}$ ${\varphi }_{1}+\beta {\mathcal{V}}+S-{\mathcalligra{m}}{\mathcal{E}}_{1}-{\mathcal{C}}_{x1}-\uptheta$ $\alpha {\mathcal{W}}-\beta {\mathcal{V}}-\updelta$	${\mathcalligra{m}}{\mathcal{E}}_{1}+\omega -S-{\mathcal{C}}_{y1}$+$\gamma L$ ${\varphi }_{1}+S-{\mathcalligra{m}}{\mathcal{E}}_{1}-{\mathcal{C}}_{x1}-\uptheta$ $-\gamma L$
	Flow in the form of policy	${\mathcalligra{m}}{\mathcal{E}}_{1}+\omega -{\mathcal{C}}_{y2}-{D}_{1}$ ${\varphi }_{1}+\beta {\mathcal{V}}-{\mathcal{C}}_{x1}-\uptheta -{\mathcalligra{m}}{\mathcal{E}}_{1}$ ${D}_{1}-\beta \mathcal{V}-\updelta$	${\varphi }_{1}+\beta \mathcal{V}-{\mathcal{C}}_{x1}-\uptheta -{\mathcalligra{m}}{\mathcal{E}}_{1}$ ${D}_{1}-\beta \mathcal{V}-\updelta$ $-{D}_{1}$

**Table 2:** Tri-party payment matrix under collaborative low-cost distribution of enterprise-alliance $(1-x).$

Behavior of game subjects		End-users	
		With low-carbon preference	Without low-carbon preference
Government	Reward and punishment combination policy	${\mathcalligra{m}}{\mathcal{E}}_{2}+A-{\mathcal{C}}_{y1}-\alpha \mathcal{W}$ ${\varphi }_{2}-A-\uptheta -{\mathcalligra{m}}{\mathcal{E}}_{2}-{\mathcal{C}}_{x2}$ $\alpha \mathcal{W}$	${\mathcalligra{m}}{\mathcal{E}}_{2}+A-{\mathcal{C}}_{y1}$+$\gamma L$ ${\varphi }_{2}-A-\uptheta -{\mathcalligra{m}}{\mathcal{E}}_{2}-{\mathcal{C}}_{x2}$ $-\gamma L$
	Flow in the form of policy	${\mathcalligra{m}}{\mathcal{E}}_{2}-{\mathcal{C}}_{y2}-{D}_{1}$ ${\varphi }_{2}-{\mathcal{C}}_{x2}-\uptheta -{\mathcalligra{m}}{\mathcal{E}}_{2}$ ${D}_{1}$	${\mathcalligra{m}}{\mathcal{E}}_{2}+{D}_{2}-{\mathcal{C}}_{y2}$ ${\varphi }_{2}-{\mathcal{C}}_{x2}-\uptheta -{\mathcalligra{m}}{\mathcal{E}}_{2}$ $-{D}_{2}$

Enterprise-alliance, end-users, and government behaviors are interactive, and the three parties utilize continuous evolution to achieve mutual benefits. In the following, the replicated dynamic equations are developed and solved for evolutionary stabilization strategies.

The payment matrices in Tables 1 and 2 show the expected benefits of collaborative low-carbon distribution and collaborative low-cost distribution for enterprise-alliance ${E}_{11}$ and ${E}_{12}$:

1 $$\begin{aligned}{E}_{11} & =\text{yz}\left({\varphi }_{1}+\beta \mathcal{V}+\text{S}-{\mathcalligra{m}}{\mathcal{E}}_{1}-{\mathcal{C}}_{x1}-\uptheta \right)+\text{y}\left(1-\text{z}\right)\left({\varphi }_{1}+\text{S}-{\mathcalligra{m}}{\mathcal{E}}_{1}-{\mathcal{C}}_{x1}-\uptheta \right)\\ &\quad+\text{z}\left(1-\text{y}\right)\left({\varphi }_{1}+\beta \mathcal{V}-{\mathcal{C}}_{x1}-\uptheta -{\mathcalligra{m}}{\mathcal{E}}_{1}\right)+\left(1-\text{z}\right)\left(1-\text{y}\right)\left({\varphi }_{1}-{\mathcal{C}}_{x1}-\uptheta -{\mathcalligra{m}}{\mathcal{E}}_{1}\right)\\ &\quad =\text{yS}+\text{z}\beta \mathcal{V}+{\varphi }_{1}-{\mathcal{C}}_{x1}-\uptheta -{\mathcalligra{m}}{\mathcal{E}}_{1}\end{aligned}$$

2 $$\begin{aligned}{E}_{12} & = \text{yz}\left({\varphi }_{2}-\text{A}-\uptheta -{\mathcalligra{m}}{\mathcal{E}}_{2}-{\mathcal{C}}_{x2}\right)+\text{y}\left(1-\text{z}\right)\left({\varphi }_{2}-\text{A}-\uptheta -{\mathcalligra{m}}{\mathcal{E}}_{2}-{\mathcal{C}}_{x2}\right)\\ & \quad +\text{z}\left(1-\text{y}\right)\left({\varphi }_{2}-{\mathcal{C}}_{x2}-\uptheta -{\mathcalligra{m}}{\mathcal{E}}_{2}\right)+\left(1-\text{z}\right)\left(1-\text{y}\right)\left({\varphi }_{2}-{\mathcal{C}}_{x2}-\uptheta -{\mathcalligra{m}}{\mathcal{E}}_{2}\right)\\ & \quad=\text{yA}+{\varphi }_{2}-{\mathcal{C}}_{x2}-\uptheta -{\mathcalligra{m}}{\mathcal{E}}_{2}\end{aligned}$$

The average expected return of the enterprise-alliance ${E}_{1}$ is:

3 $${E}_{1}=x{E}_{11}+\left(1-x\right){E}_{12}$$

This leads to the following equation for the replication dynamics of the enterprise-alliance ${F}_{\left(x\right)}$ as^34^:

4 $${F}_{\left(x\right)}=\frac{{d}_{x}}{{d}_{t}}=x\left({E}_{11}{-E}_{1}\right)=x\left(1-x\right)\left({E}_{11}-{E}_{12}\right)=x\left(1-x\right)\left[\text{y}\left(\text{S}-\text{A}\right)+\left({\varphi }_{1}-{\varphi }_{2}\right)+\left({\mathcal{C}}_{x2}-{\mathcal{C}}_{x1}\right)+{\mathcalligra{m}}\left({\mathcal{E}}_{2}-{\mathcal{E}}_{1}\right)+\text{z}\beta \mathcal{V}\right]$$

Similarly, it follows that the expected returns to the government's adoption of the policy of reward and punishment combination and the policy of flow in the form ${E}_{21}$ and ${E}_{22}$ respectively:

5 $$\begin{aligned}{E}_{21} & = x\text{z}\left({\mathcalligra{m}}{\mathcal{E}}_{1}+\omega -\text{S}-{\mathcal{C}}_{\text{y}1}-\alpha \mathcal{W}\right)+x\left(1-\text{z}\right)\left({\mathcalligra{m}}{\mathcal{E}}_{1}+\omega -\text{S}-{\mathcal{C}}_{\text{y}1}+\gamma L\right)\\ & \quad +\text{z}\left(1-x\right)\left({\mathcalligra{m}}{\mathcal{E}}_{2}+\text{A}-{\mathcal{C}}_{\text{y}1}-\alpha \mathcal{W}\right)+\left(1-\text{z}\right)\left(1-x\right)\left({\mathcalligra{m}}{\mathcal{E}}_{2}+\text{A}-{\mathcal{C}}_{\text{y}1}+\gamma L\right)\\ & \quad =x\left[{\mathcalligra{m}}\left({\mathcal{E}}_{1}-{\mathcal{E}}_{2}\right)+\omega -\text{S}-\text{A}\right]+{\mathcalligra{m}}{\mathcal{E}}_{2}+\text{A}-{\mathcal{C}}_{\text{y}1}+\gamma L-\text{z}\left(\alpha \mathcal{W}+\gamma L\right)\end{aligned}$$

6 $$\begin{aligned}{E}_{22} & = x\text{z}\left({\mathcalligra{m}}{\mathcal{E}}_{1}+\omega -{\mathcal{C}}_{\text{y}2}-{D}_{1}\right)+x\left(1-\text{z}\right)\left({\mathcalligra{m}}{\mathcal{E}}_{1}+\omega -{\mathcal{C}}_{\text{y}2}+{D}_{2}\right)+\text{z}\left(1-x\right)\left({\mathcalligra{m}}{\mathcal{E}}_{2}-{\mathcal{C}}_{\text{y}2}-{D}_{1}\right)\\ & \quad +\left(1-\text{z}\right)\left(1-x\right)\left({\mathcalligra{m}}{\mathcal{E}}_{2}-{\mathcal{C}}_{\text{y}2}+{D}_{2}\right)\\ &\quad=x\left[{\mathcalligra{m}}\left({\mathcal{E}}_{1}-{\mathcal{E}}_{2}\right)+\omega \right]+{\mathcalligra{m}}{\mathcal{E}}_{2}-{\mathcal{C}}_{\text{y}2}+{D}_{2}-\text{z}\left({D}_{1}+{D}_{2}\right)\end{aligned}$$

The average expected return to the government ${E}_{2}$ is:

7 $${E}_{2}=\text{y}{E}_{21}+\left(1-\text{y}\right){E}_{22}$$

This leads to the following equation for the replication dynamics of the government ${F}_{\left(y\right)}$ as:

8 $$\begin{aligned}{F}_{\left(y\right)} & = \frac{{d}_{y}}{{d}_{t}}=y\left({E}_{21}{-E}_{2}\right)=y\left(1-y\right)\left({E}_{21}-{E}_{22}\right)\\ &=y\left(1-y\right)\left[\left({\mathcal{C}}_{y2}-{\mathcal{C}}_{y1}\right)-x\left(S+A\right) +A+\gamma L-{D}_{2}+z\left({D}_{1}+{D}_{2}-\alpha \mathcal{W}-\gamma L\right)\right]\end{aligned}$$

Similarly, the expected returns of the end-users with and without low-carbon preference are ${E}_{31}$ and ${E}_{32}$ respectively:

9 $$\begin{aligned}{E}_{31} & = xy\left(\alpha \mathcal{W}-\beta \mathcal{V}-\delta \right)+x\left(1-y\right)\left({D}_{1}-\beta \mathcal{V}-\delta \right)+y\alpha \mathcal{W}\left(1-x\right)\\ & \quad+{D}_{1}\left(1-x\right)\left(1-y\right)=y\left(\alpha \mathcal{W}-{D}_{1}\right)-x\left(\beta \mathcal{V}+\delta \right)+{D}_{1}\end{aligned}$$

10 $${E}_{32}=-xy\gamma L-x{D}_{2}\left(1-y\right)-y\gamma L\left(1-x\right)-{D}_{1}\left(1-x\right)\left(1-y\right)=y\left({D}_{2}-\gamma L\right)-{D}_{2}$$

The average expected return to the end-users ${E}_{3}$ is:

11 $${E}_{3}=\text{z}{E}_{31}+\left(1-\text{z}\right){E}_{32}$$

This leads to the following equation for the dynamics of end-users replication ${F}_{\left(z\right)}$ as:

12 $${F}_{\left(z\right)}=\frac{{d}_{z}}{{d}_{t}}=z\left({E}_{31}{-E}_{3}\right)=z\left(1-z\right)\left({E}_{31}-{E}_{32}\right)=z\left(1-z\right)\left[y\left(\alpha \mathcal{W}+\gamma L-{D}_{1}-{D}_{2}\right)-x\left(\beta \mathcal{V}+\delta \right)+{D}_{1}+{D}_{2}\right]$$

## Game model solving and analysis

### Analysis of the stability of single subject strategies

#### Strategic stability analysis of enterprise-alliance

From the above, the replication dynamic equation of the corporate alliance game strategy is Eq. (4):

$${F}_{\left(x\right)}=x\left(1-x\right)\left[\text{y}\left(\text{S}-\text{A}\right)+\left({\varphi }_{1}-{\varphi }_{2}\right)+\left({\mathcal{C}}_{x2}-{\mathcal{C}}_{x1}\right)+{\mathcalligra{m}}\left({\mathcal{E}}_{2}-{\mathcal{E}}_{1}\right)+\text{z}\beta \mathcal{V}\right]$$

13 $$\text{set up }{H}_{\left(\text{y}\right)}=\text{y}\left(\text{S}-\text{A}\right)+\left({\varphi }_{1}-{\varphi }_{2}\right)+\left({\mathcal{C}}_{x2}-{\mathcal{C}}_{x1}\right)+{\mathcalligra{m}}\left({\mathcal{E}}_{2}-{\mathcal{E}}_{1}\right)+\text{z}\beta \mathcal{V}$$

At this point the first order derivative of $x$ the first-order derivative of ${\mathcalligra{d}}\left({F}_{\left(x\right)}\right)/{\mathcalligra{d}}x=\left(1-2x\right){H}_{\left(\text{y}\right)}$

14 $$\text{Order }\widehat{y}=\frac{\left({\varphi }_{1}-{\varphi }_{2}\right)+\left({\mathcal{C}}_{x2}-{\mathcal{C}}_{x1}\right)+{\mathcalligra{m}}\left({\mathcal{E}}_{2}-{\mathcal{E}}_{1}\right)+\text{z}\beta \mathcal{V}}{\left(\text{A}-\text{S}\right)}$$

1. When $\text{y}=\widehat{\text{y}}$ and ${F}_{\left(x\right)}=0$, regardless of whether $x$ takes any value, the enterprise-alliance ' strategy tends to stabilize, which indicates that the enterprise-alliance is a stable strategy regardless of what strategy it chooses^34^, as shown in Fig. 3a.

**Figure 3:**
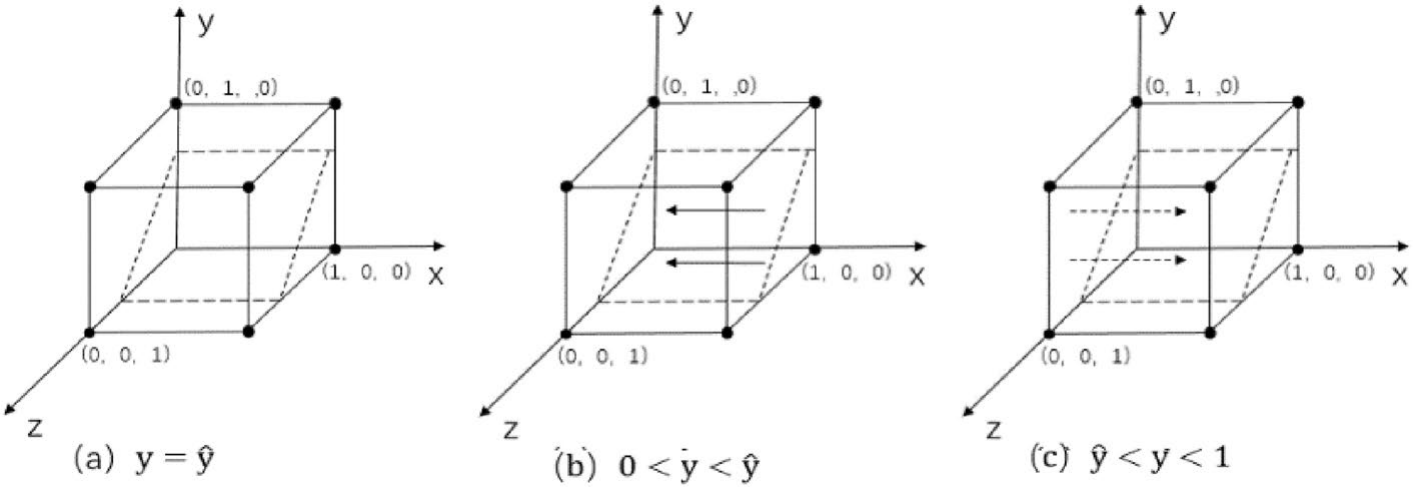
Phase diagram of replication dynamics for enterprise-alliance.
2. When $\text{y}\ne \widehat{\text{y}}$, order ${F}_{\left(x\right)}=0$ , there exist two equilibrium points:${x}_{1}^{*}=0$ and ${x}_{2}^{*}=1$ .According to the stability theorem of differential equations, realizing the stabilization needs to satisfy both ${F}_{\left(x\right)}=0$ and ${\mathcalligra{d}}\left({F}_{\left(x\right)}\right)/{\mathcalligra{d}}x<0$.${\mathcalligra{d}}{H}_{\left(\text{y}\right)}/{\mathcalligra{d}}\text{y}=\text{S}-\text{A}>0$ and ${H}_{\left(\text{y}\right)}$ is an increasing function.

When $0<\text{y}<\widehat{\text{y}}$ and ${H}_{\left(\text{y}\right)}<0$,${\mathcalligra{d}}\left({F}_{\left(x\right)}\right)/{\mathcalligra{d}}x{|}_{{x}_{1}^{*}=0}<0$,$\left({F}_{\left(x\right)}\right)/{\mathcalligra{d}}x{|}_{{x}_{1}^{*}=1}>0$.Therefore ${x}_{1}^{*}=0$ is an evolutionarily stable strategy. The adoption of collaborative low-cost distribution by the enterprise-alliance is the only evolutionary stable strategy globally, as shown in Fig. 3b. Similarly, when $\widehat{\text{y}}<\text{y}<1$,$\left({F}_{\left(x\right)}\right)/{\mathcalligra{d}}x{|}_{{x}_{2}^{*}=1}<0$ , and ${x}_{2}^{*}=1$ is the stable strategy, the enterprise-alliance adopts collaborative low-carbon distribution as the global unique evolutionary stable strategy as shown in Fig. 3c.

#### Strategic stability analysis of government

From the above, the replication dynamic equation for the government game strategy is:

$${F}_{\left(y\right)}=\text{y}\left(1-\text{y}\right)\left[\left({\mathcal{C}}_{\text{y}2}-{\mathcal{C}}_{\text{y}1}\right)-x\left(\text{S}+\text{A}\right)+\text{A}+\gamma L-{D}_{2}+\text{z}\left({D}_{1}+{D}_{2}-\alpha \mathcal{W}-\gamma L\right)\right]$$

15 $$\text{Set up }{G}_{\left(\text{z}\right)}=\left({\mathcal{C}}_{\text{y}2}-{\mathcal{C}}_{\text{y}1}\right)-x\left(\text{S}+\text{A}\right)+\text{A}+\gamma L-{D}_{2}+\text{z}\left({D}_{1}+{D}_{2}-\alpha \mathcal{W}-\gamma L\right)$$

At this point the first order derivative of $\text{y}$ the first-order derivative of ${\mathcalligra{d}}\left({F}_{\left(\text{y}\right)}\right)/{\mathcalligra{d}}\text{y}=\left(1-2\text{y}\right){G}_{\left(\text{z}\right)}$

16 $$\text{Order }\widehat{z}=\frac{\left({\mathcal{C}}_{y2}-{\mathcal{C}}_{y1}\right)-x\left(S+A\right)+A+\gamma L-{D}_{2}}{\alpha \mathcal{W}+\gamma L-{D}_{1}-{D}_{2}}$$

1. When $\text{z}=\widehat{\text{z}}$ and ${F}_{\left(\text{y}\right)}=0$ ,regardless of whether $\text{y}$ takes any value, the government's strategy tends to stabilize, which indicates that the government's strategy is stable regardless of what it chooses, as shown in Fig. 4a.

**Figure 4:**
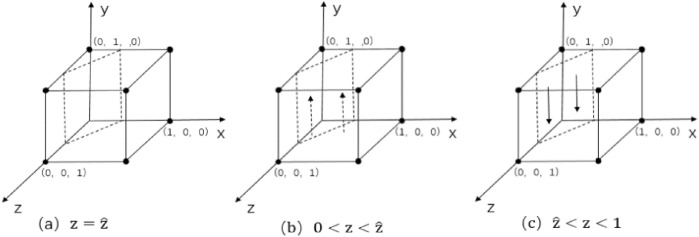
Phase diagram of replication dynamics for government.
2. When $\text{z}\ne \widehat{\text{z}}$, order ${F}_{\left(\text{y}\right)}=0$ , there exist two equilibrium points:${\text{y}}_{1}^{*}=0$ and ${\text{y}}_{2}^{*}=1$.According to the stability theorem of differential equations, realizing the strategy stabilization needs to satisfy both ${F}_{\left(\text{y}\right)}=0$ and ${\mathcalligra{d}}\left({F}_{\left(\text{y}\right)}\right)/{\mathcalligra{d}}\text{y}<0$.${\mathcalligra{d}}{G}_{\left(\text{z}\right)}/{\mathcalligra{d}}\text{z}={D}_{1}+{D}_{2}-\alpha \mathcal{W}-\gamma L<0$ and ${G}_{\left(\text{z}\right)}$ is a decreasing function.

When $0<\text{z}<\widehat{\text{z}}$ and ${G}_{\left(\text{z}\right)}>0$,${\mathcalligra{d}}\left({F}_{\left(\text{y}\right)}\right)/{\mathcalligra{d}}\text{y}{|}_{{\text{y}}_{1}^{*}=0}>0$,$\left({F}_{\left(\text{y}\right)}\right)/{\mathcalligra{d}}\text{y}{|}_{{\text{y}}_{2}^{*}=1}<0$, therefore ${\text{y}}_{2}^{*}=1$ is an evolutionarily stabilization strategy. The government adopts the flow in the form policy as the only evolutionary stabilization strategy in the whole world, as shown in Fig. 4b. Similarly, when $\widehat{\text{z}}<\text{z}<1$ and $\left({F}_{\left(\text{y}\right)}\right)/{\mathcalligra{d}}\text{y}{|}_{{\text{y}}_{1}^{*}=0}<0$,${\text{y}}_{1}^{*}=0$ is an evolutionarily stable strategy. The government adopts the policy of reward and punishment combination as the only evolutionary stable strategy in the whole situation, as shown in Fig. 4c.

#### Strategy stability analysis for end-users

From the above, the replication dynamic equation for the end-user game strategy is:

$${F}_{\left(\text{z}\right)}=\text{z}\left(1-\text{z}\right)\left[y\left(\alpha \mathcal{W}+\gamma L-{D}_{1}-{D}_{2}\right)-x\left(\beta \mathcal{V}+\delta \right)+{D}_{1}+{D}_{2}\right]$$

17 $$\text{set up }{R}_{\left(x\right)}=y\left(\alpha \mathcal{W}+\gamma L-{D}_{1}-{D}_{2}\right)-x\left(\beta \mathcal{V}+\delta \right)+{D}_{1}+{D}_{2}$$

At this point the first order derivative of $\text{z}$ the first-order derivative of ${\mathcalligra{d}}\left({F}_{\left(\text{z}\right)}\right)/{\mathcalligra{d}}\text{z}=\left(1-2\text{z}\right){R}_{\left(x\right)}$

18 $$\text{Order }\widehat{x}=\frac{y\left(\alpha \mathcal{W}+\gamma L-{D}_{1}-{D}_{2}\right)+{D}_{1}+{D}_{2}}{\beta \mathcal{V}+\delta }$$

1. When $x=\widehat{x}$ and ${F}_{\left(\text{z}\right)}=0$ , regardless of whether $\text{z}$ takes any value, the end-users' strategy tends to stabilize, which indicates that the end-users' strategy is a stable strategy regardless of the strategy chosen, as shown in Fig. 5a.

**Figure 5:**
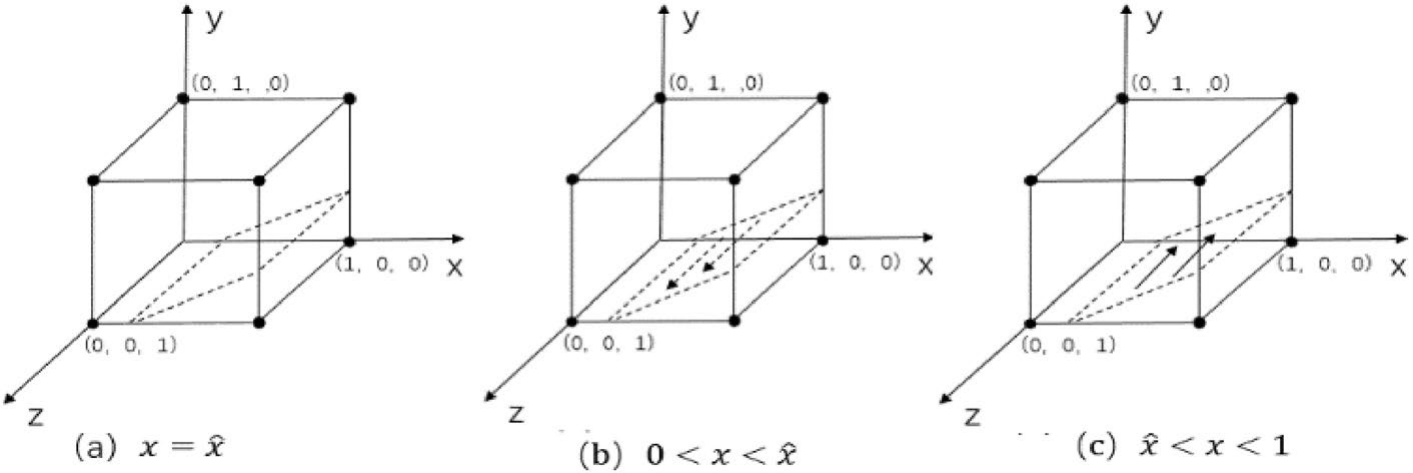
Phase diagram of replication dynamics for end-users'.
2. When $x\ne \widehat{x} ,\text{order}{F}_{\left(\text{z}\right)}=0$, there exist two equilibrium points:${\text{ z}}_{1}^{*}=0$ and ${\text{z}}_{2}^{*}=1$. According to the stability theorem of differential equations, realizing strategy stabilization needs to satisfy both ${F}_{\left(\text{z}\right)}=0$ and ${\mathcalligra{d}}\left({F}_{\left(\text{z}\right)}\right)/{\mathcalligra{d}}\text{z}<0$ . Since ${\mathcalligra{d}} {R}_{\left(x\right)}/{\mathcalligra{d}}x=-\left(\beta \mathcal{V}+\delta \right)<0$, ${R}_{\left(x\right)}$ is a decreasing function.

When $$0<x<\widehat{x}$$ and $${R}_{\left(x\right)}>0$$ ,$${\mathcalligra{d}}\left({F}_{\left(\text{z}\right)}\right)/{\mathcalligra{d}}\text{z}{|}_{{\text{z}}_{1}^{*}=0}>0$$ ,$$\left({F}_{\left(\text{z}\right)}\right)/{\mathcalligra{d}}\text{z}{|}_{{\text{z}}_{2}^{*}=1}<0$$

Therefore ${\text{z}}_{2}^{*}=1$ is an evolutionarily stable strategy. End-users without low-carbon preference is the only evolutionarily stable strategy globally, as shown in Fig. 5b. Similarly, when $\widehat{x}<x<1$ and $\left({F}_{\left(\text{z}\right)}\right)/{\mathcalligra{d}}\text{z}{|}_{{\text{z}}_{1}^{*}=0}<0$ , the ${\text{z}}_{1}^{*}=0$ is an evolutionarily stable strategy. End-users with low-carbon preference is the global unique evolutionary stable strategy, as shown in Fig. 5c.

### Stability analysis of the equilibrium point of the tripartite evolutionary game system

#### Three-party game model ESS solving

The previous section analyzed the equilibrium conditions for each game subject to reach the stabilization strategy from the perspective of a single game subject, but in essence, the achievement of the final stable state of the system requires the joint action of the three parties^35^. Therefore, in this section, the asymptotic stability of the equilibrium point is determined by constructing a Jacobi matrix and solving for the eigenvalues, taking into account the existing studies^36,37^. According to the replicated dynamic equations ${F}_{\left(x\right) },{F}_{\left(y\right)}$ and ${F}_{\left(\text{z}\right)}$ ,the of $x$,$y$ and $\text{z}$ derivation yield the Jacobian matrix^38^ . In fact, let the replicated dynamic equation of the three parties of interest be 0. Eight pure strategy Nash equilibrium points can be obtained:$\left(0,0,0\right)\left(0,0,1\right)\left(0,1,0\right)\left(1,0,0\right)\left(1,1,0\right)\left(1,0,1\right)\left(0,1,1\right)\left(1,1,1\right)$.The Jacobi matrix is obtained from the above replicated dynamic equation as shown in Eq. (19):

19 $$\text{J}=\left[\begin{array}{ccc}\frac{\partial {F}_{\left(x\right)}}{\partial x}& \frac{\partial {F}_{\left(x\right)}}{\partial y}& \frac{\partial {F}_{\left(x\right)}}{\partial \text{z}}\\ \frac{\partial {F}_{\left(y\right)}}{\partial x}& \frac{\partial {F}_{\left(y\right)}}{\partial y}& \frac{\partial {F}_{\left(y\right)}}{\partial \text{z}}\\ \frac{\partial {F}_{\left(\text{z}\right)}}{\partial x}& \frac{\partial {F}_{\left(\text{z}\right)}}{\partial y}& \frac{\partial {F}_{\left(\text{z}\right)}}{\partial \text{z}}\end{array}\right]$$

Among them:

20 $$\frac{\partial {F}_{\left(x\right)}}{\partial x}=\left(1-2x\right)\left[\text{y}\left(\text{S}-\text{A}\right)+\left({\varphi }_{1}-{\varphi }_{2}\right)+\left({\mathcal{C}}_{x2}-{\mathcal{C}}_{x1}\right)+{\mathcalligra{m}}\left({\mathcal{E}}_{2}-{\mathcal{E}}_{1}\right)+\text{z}\beta \mathcal{V}\right]$$

21 $$\frac{\partial {F}_{\left(x\right)}}{\partial y}=x\left(1-x\right)\left(\text{S}-\text{A}\right)$$

22 $$\frac{\partial {F}_{\left(x\right)}}{\partial z}=\beta \mathcal{V}x\left(1-x\right)$$

23 $$\frac{\partial {F}_{\left(y\right)}}{\partial x}=-y\left(1-y\right)\left(S+A\right)$$

24 $$\frac{\partial {F}_{\left(y\right)}}{\partial y}=\left(1-2y\right)\left[\left({\mathcal{C}}_{y2}-{\mathcal{C}}_{y1}\right)-x\left(S+A\right)+A+\gamma L-{D}_{2}+z\left({D}_{1}+{D}_{2}-\alpha \mathcal{W}-\gamma L\right)\right]$$

25 $$\frac{\partial {F}_{\left(y\right)}}{\partial z}=\left({D}_{1}+{D}_{2}-\alpha \mathcal{W}-\gamma L\right)$$

26 $$\frac{\partial {F}_{\left(z\right)}}{\partial x}=-z\left(1-z\right)\left(\beta \mathcal{V}+\delta \right)$$

27 $$\frac{\partial {F}_{\left(z\right)}}{\partial y}=z\left(1-z\right)\left(\alpha \mathcal{W}+\gamma L-{D}_{1}-{D}_{2}\right)$$

28 $$\frac{\partial {F}_{\left(z\right)}}{\partial z}=\left(1-2z\right)\left[y\left(\alpha \mathcal{W}+\gamma L-{D}_{1}-{D}_{2}\right)-x\left(\beta \mathcal{V}+\delta \right)+{D}_{1}+{D}_{2}\right]$$

#### Equilibrium point stability analysis

From evolutionary game theory^39^ , the stability of the equilibrium point of the system can be judged by the eigenvalues of the Jacobi matrix proposed by Friedman^40^. According to Lyapunov’s^41,42^ first method, when the eigenvalues are negative, the equilibrium point is stable; when the eigenvalues are positive, the equilibrium point is unstable. The above eight equilibrium points are substituted into the Jacobi matrix to obtain the corresponding eigenvalues, as shown in the following table:

The local equilibrium points in the table are stable points if the three corresponding eigenvalues are not greater than zero. By further solving the eigenvalues in Table 3, we find that there are six stable points in the evolutionary game, and the specific scenarios are as follows:

**Table 3:** Stability analysis of equilibrium points.

Balance point	Eigenvalues ${\lambda }_{1}$ ,${\lambda }_{2}$ ,${\lambda }_{3}$	Real symbol
$\left(0,0,0\right)$	$\left({\varphi }_{1}-{\varphi }_{2}\right)+\left({\mathcal{C}}_{x2}-{\mathcal{C}}_{x1}\right)+{\mathcalligra{m}}\left({\mathcal{E}}_{2}-{\mathcal{E}}_{1}\right)$ ${\mathcal{C}}_{y2}-{\mathcal{C}}_{y1}+A+\gamma L-{D}_{2}$ ${D}_{1}+{D}_{2}$	(*, *, +)
$\left(0,0,1\right)$	$\left({\varphi }_{1}-{\varphi }_{2}\right)+\left({\mathcal{C}}_{x2}-{\mathcal{C}}_{x1}\right)+{\mathcalligra{m}}\left({\mathcal{E}}_{2}-{\mathcal{E}}_{1}\right)+\beta \mathcal{V}$ ${\mathcal{C}}_{y2}-{\mathcal{C}}_{y1}+A+{D}_{1}-\alpha \mathcal{W}$ $-\left({D}_{1}+{D}_{2}\right)$	(*, *, -)
$\left(0,1,0\right)$	$\left(\text{S}-\text{A}\right)+\left({\varphi }_{1}-{\varphi }_{2}\right)+\left({\mathcal{C}}_{x2}-{\mathcal{C}}_{x1}\right)+{\mathcalligra{m}}\left({\mathcal{E}}_{2}-{\mathcal{E}}_{1}\right)$ $-\left({\mathcal{C}}_{y2}-{\mathcal{C}}_{y1}+A+\gamma L-{D}_{2}\right)$ $\alpha \mathcal{W}+\gamma L$	(*, *, +)
$\left(1,0,0\right)$	$-\left[\left({\varphi }_{1}-{\varphi }_{2}\right)+\left({\mathcal{C}}_{x2}-{\mathcal{C}}_{x1}\right)+{\mathcalligra{m}}\left({\mathcal{E}}_{2}-{\mathcal{E}}_{1}\right)\right]$ ${\mathcal{C}}_{y2}-{\mathcal{C}}_{y1}-S+\gamma L-{D}_{2}$ ${D}_{1}+{D}_{2}-\left(\beta \mathcal{V}+\delta \right)$	(*, *, *)
$\left(1,1,0\right)$	$-\left[\left(\text{S}-\text{A}\right)+\left({\varphi }_{1}-{\varphi }_{2}\right)+\left({\mathcal{C}}_{x2}-{\mathcal{C}}_{x1}\right)+{\mathcalligra{m}}\left({\mathcal{E}}_{2}-{\mathcal{E}}_{1}\right)\right]$ $-\left({\mathcal{C}}_{y2}-{\mathcal{C}}_{y1}-S+\gamma L-{D}_{2}\right)$ $\alpha \mathcal{W}+\gamma L-\left(\beta \mathcal{V}+\delta \right)$	(*, *, *)
$\left(1,0,1\right)$	$-\left[\left({\varphi }_{1}-{\varphi }_{2}\right)+\left({\mathcal{C}}_{x2}-{\mathcal{C}}_{x1}\right)+{\mathcalligra{m}}\left({\mathcal{E}}_{2}-{\mathcal{E}}_{1}\right)+\beta \mathcal{V}\right]$ ${\mathcal{C}}_{y2}-{\mathcal{C}}_{y1}-S+{D}_{1}-\alpha \mathcal{W}$ $\left(\beta \mathcal{V}+\delta \right)-\left({D}_{1}+{D}_{2}\right)$	(*, *, *)
$\left(0,1,1\right)$	$\left(\text{S}-\text{A}\right)+\left({\varphi }_{1}-{\varphi }_{2}\right)+\left({\mathcal{C}}_{x2}-{\mathcal{C}}_{x1}\right)+{\mathcalligra{m}}\left({\mathcal{E}}_{2}-{\mathcal{E}}_{1}\right)+\beta \mathcal{V}$ $-\left({\mathcal{C}}_{y2}-{\mathcal{C}}_{y1}+A+{D}_{1}-\alpha \mathcal{W}\right)$ $-\left(\alpha \mathcal{W}+\gamma L\right)$	(*, *, -)
$\left(1,1,1\right)$	$-\left[\left(\text{S}-\text{A}\right)+\left({\varphi }_{1}-{\varphi }_{2}\right)+\left({\mathcal{C}}_{x2}-{\mathcal{C}}_{x1}\right)+{\mathcalligra{m}}\left({\mathcal{E}}_{2}-{\mathcal{E}}_{1}\right)+\beta \mathcal{V}\right]$ $-\left({\mathcal{C}}_{y2}-{\mathcal{C}}_{y1}-S+{D}_{1}-\alpha \mathcal{W}\right)$ $\left(\beta \mathcal{V}+\delta \right)-\left(\alpha \mathcal{W}+\gamma L\right)$	(*, *, *)

" + " indicates that the eigenvalue is positive, "−" indicates that the eigenvalue is negative, and "*" indicates that the eigenvalue is positive or negative depending on the specific parameter value.

Scenario 1 $\left(1,1,1\right)$ : When $\left(S-A\right)+\left({\mathcal{C}}_{x2}-{\mathcal{C}}_{x1}\right)+{\mathcalligra{m}}\left({\mathcal{E}}_{2}-{\mathcal{E}}_{1}\right)+\beta \mathcal{V}>{\varphi }_{2}-{\varphi }_{1}$ ,${\mathcal{C}}_{y1}-{\mathcal{C}}_{y2}<{D}_{1}-\alpha \mathcal{W}-S$ and $\beta \mathcal{V}+\delta <\alpha \mathcal{W}+\gamma L$ ,the stable strategy of the evolutionary game is (collaborative low-carbon distribution, reward and punishment combination, with low-carbon preference). In this scenario, the benefits of the enterprise-alliance when it engages in low-carbon distribution are supplemented by the government and end-users, and the benefits of collaborative low-carbon distribution are much greater than the benefits of collaborative low-cost distribution, so the enterprise-alliance group will choose to engage in collaborative low-carbon distribution. The net benefit^43^ to the government of adopting the policy of reward and punishment combination is greater than the net benefit of adopting flow in the form of policy, so the government group will choose to adopt policy of reward and punishment combination to guide the enterprise-alliance and the end-users, and the government can obtain the greatest environmental benefits. The benefits to end-users with low-carbon preference outweigh the costs, so the end-users will gradually support low-carbon distribution through enterprise-alliance.

Scenario 2 $\left(0,1,1\right)$ : When $\left(S-A\right)+\left({\mathcal{C}}_{x2}-{\mathcal{C}}_{x1}\right)+{\mathcalligra{m}}\left({\mathcal{E}}_{2}-{\mathcal{E}}_{1}\right)+\beta \mathcal{V}<{\varphi }_{2}-{\varphi }_{1}$ , ${\mathcal{C}}_{y1}-{\mathcal{C}}_{y2}<A-{D}_{1}-\alpha \mathcal{W}$ , the evolutionary game stabilizes the strategy as (collaborative low-cost distribution, reward-punishment combination policy, with low-carbon preference). In this scenario, the enterprise-alliance group will prefer collaborative low-cost distribution because the revenue subsidies to the enterprise-alliance from the government and end-users are smaller than the revenue difference between the enterprise-alliance performing low-cost distribution and low-carbon distribution. The government will prefer reward and punishment combination policy because the government receives a penalty for low-carbon distribution, which is greater than the difference between the government's costs of adopting the two policies.

Scenario 3 $\left(1,0,1\right)$ : When $\left({\mathcal{C}}_{x2}-{\mathcal{C}}_{x1}\right)+{\mathcalligra{m}}\left({\mathcal{E}}_{2}-{\mathcal{E}}_{1}\right)+\beta \mathcal{V}>{\varphi }_{2}-{\varphi }_{1}$,$S+{D}_{1}-\alpha \mathcal{W}<{\mathcal{C}}_{y1}-{\mathcal{C}}_{y2}$ and $\beta \mathcal{V}+\delta <{D}_{1}+{D}_{2}$,the stable strategy of the evolutionary game is (collaborative low-carbon distribution, flow in the form of policy, with low-carbon preference). In this scenario, although the enterprise-alliance does not have government incentives, the end-users with low-carbon preference is willing to pay a certain distribution cost. The income of the enterprise-alliance's collaborative low-carbon distribution is greater than income of the collaborative low-cost distribution, so the enterprise-alliance group still chooses the collaborative low-carbon distribution. When the government adopts flow in the form of policy, the incentives for end-users are greater than the costs that end-users with low-carbon preference need to pay, so the end-users group will gradually have low-carbon preference and support the enterprise-alliance in collaborating on low-carbon distribution.

Scenario 4 $\left(1,1,0\right)$ : When $\left(\text{S}-\text{A}\right)+\left({\mathcal{C}}_{x2}-{\mathcal{C}}_{x1}\right)+{\mathcalligra{m}}\left({\mathcal{E}}_{2}-{\mathcal{E}}_{1}\right)<{\varphi }_{2}-{\varphi }_{1}$,$S+\gamma L-{D}_{2}>{\mathcal{C}}_{y1}-{\mathcal{C}}_{y2}$,$\text{and }\alpha \mathcal{W}+\gamma L<\beta \mathcal{V}+\delta$ , the stable strategy of the evolutionary game is (collaborative low-carbon distribution, reward and punishment combination policy, without low-carbon preference). In this scenario, the reward received by the end-users are less than the costs they need to bear to support low-carbon distribution, so the end-users group's low-carbon preference will gradually decrease until it disappears. Although the enterprise-alliance does not support of end-users, the benefit of collaborative low-carbon distribution is still greater than benefit of collaborative low-cost distribution under the government subsidy, so the enterprise-alliance group will choose collaborative low-carbon distribution. Similarly, the benefit to the government of adopting reward and punishment combination policy is greater than benefit of adopting flow in the form of policy, so the government group will choose to adopt policy of reward and punishment combination.

Scenario 5 $\left(1,0,0\right)$ : When $\left({\mathcal{C}}_{x2}-{\mathcal{C}}_{x1}\right)+{\mathcalligra{m}}\left({\mathcal{E}}_{2}-{\mathcal{E}}_{1}\right)>{\varphi }_{2}-{\varphi }_{1}$,$\gamma L-S-{D}_{2}<{\mathcal{C}}_{y1}-{\mathcal{C}}_{y2}$,$\text{and }{D}_{1}+{D}_{2}<\beta \mathcal{V}+\delta$ , the evolutionary game stabilizes the strategy (collaborative low-carbon distribution, flow in the form of policy, without low- carbon preference). In this scenario, the government subsidy to end-users is less than their cost of supporting low-carbon distribution, so the end-users group's low-carbon preference fades. The benefit to the enterprise-alliance of undertaking collaborative low-carbon distribution is greater than benefit of undertaking collaborative low-cost distribution, so the enterprise-alliance group will undertake collaborative low-carbon distribution.

Scenario 6 $\left(0,0,1\right)$ : When $\left({\mathcal{C}}_{x2}-{\mathcal{C}}_{x1}\right)+{\mathcalligra{m}}\left({\mathcal{E}}_{2}-{\mathcal{E}}_{1}\right)+\beta \mathcal{V}<{\varphi }_{2}-{\varphi }_{1}$,$A+{D}_{1}-\alpha \mathcal{W}<{\mathcal{C}}_{y1}-{\mathcal{C}}_{y2}$,the stable strategy of the evolutionary game is (collaborative low-cost distribution, flow in the form of policy, with low-carbon preference). In this scenario, although the end-users have low-carbon preference, the cost they are willing to pay is lower, and the enterprise-alliance adopts the flow in the form of policy, therefore the enterprise-alliance group will choose the collaborative low-cost distribution based on the principle of profit maximization. The benefits to the government of adopting reward and punishment combination policy are lower than those of adopting flow in the form of policy, so the government group will also choose to adopt flow in the form of policy.

## Data simulation

To verify the validity of the evolutionary stability analysis, more intuitively present the evolutionary stability process and explore the influence of relevant factors on the strategic behavior of each subject, this paper uses MATLAB R2021 (Full name of software and Version number: Matlab R2021a Win64 Crack. URL link: https://matlab.waltzsy.com/) software to carry out sensitivity analysis and numerical simulation on the equilibrium strategies of each subject^44^. At the same time, the parameters involved in the replicated dynamic equation system assumed to meet the corresponding conditions of different evolutionary stable states, to make them as close as possible to the actual case situation and to representatively present the model's evolution of the game laws and characteristics^45,46^.

**Figure 6:**
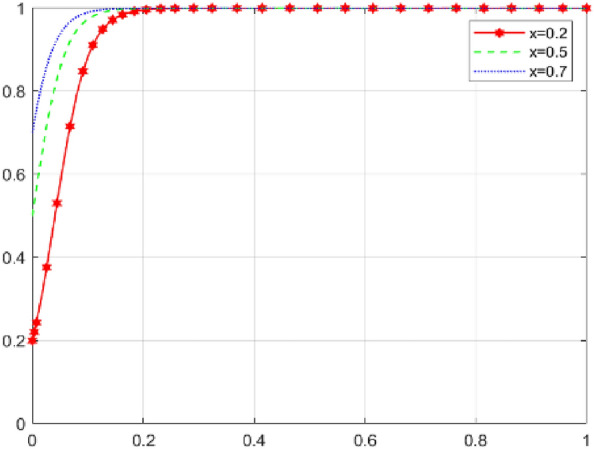
Evolutionary game diagram with different times for x.

**Figure 7:**
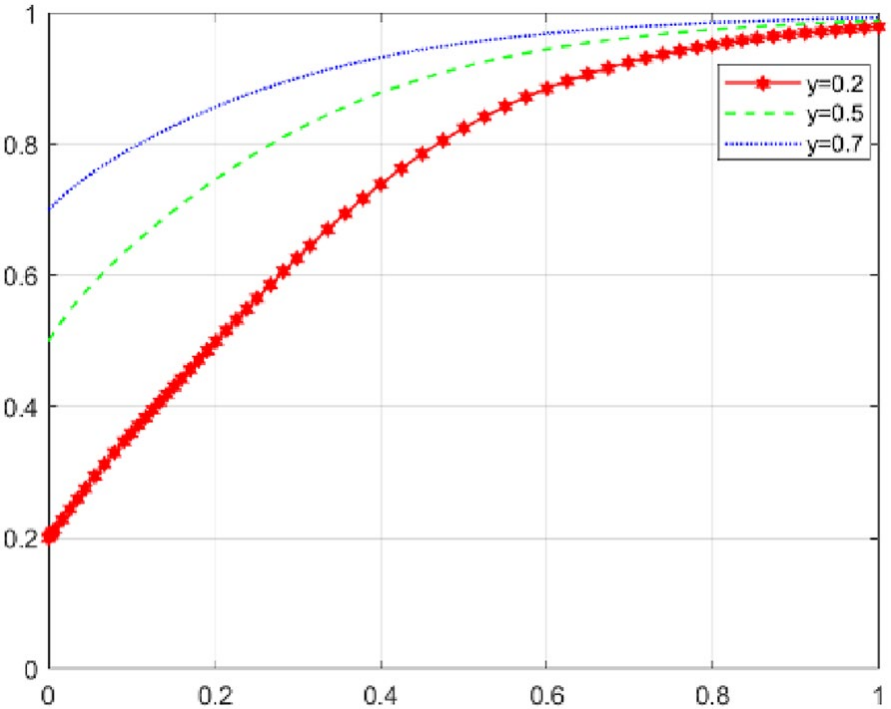
Evolutionary game diagram with different times for y.

**Figure 8:**
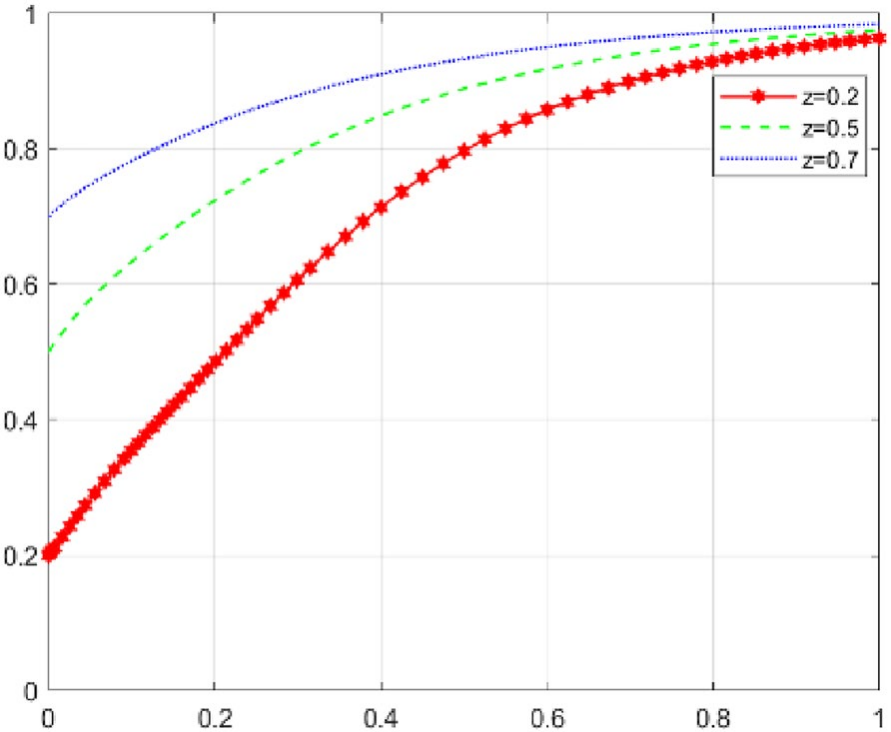
Evolutionary game diagram with different times for z.

First, assume that array 1 satisfies Case 1: ${\mathcal{C}}_{x1}$=*50，*${\mathcal{C}}_{x2}$=*40，*${\mathcal{E}}_{1}=50$* ，*${\mathcal{E}}_{2}=35$*，*${\varphi }_{1}=300$*，*${\varphi }_{2}=250$*，*${\mathcalligra{m}}=0.5$*，*$S=5$*，*$A=1$*，*${\mathcal{C}}_{y1}=15$*，*${\mathcal{C}}_{y2}=20$*，*$\alpha =0.1$*，*$\mathcal{W}=10$*，*$\gamma =0.7$*，*$L=10$*，*${D}_{1}=4$*，*${D}_{2}=1$*，*$\beta =0.5$*，*$\mathcal{V}=6$*，*$\delta =1$. The array 1 $,x$,$y$ and $\text{z}$ evolutionary simulation routes under different initial value states are explored to verify the validity of the parameters. The initial values of $x$,$y$ and $\text{z}$ are set to 0.2, 0.5, and 0.7, respectively, and the results are shown in Figs. 6, 7 and 8: according to the model results, the initial values of $x$,$y$,$\text{z}$ converge to 1 in the end, which is consistent with the evolutionary equilibrium point of case 1 $\left(1,1,1\right)$ , therefore the model and parameters are valid.

### Analysis of the impact of different strategy choices of multiple subjects on the outcome of the evolutionary game

**Figure 9:**
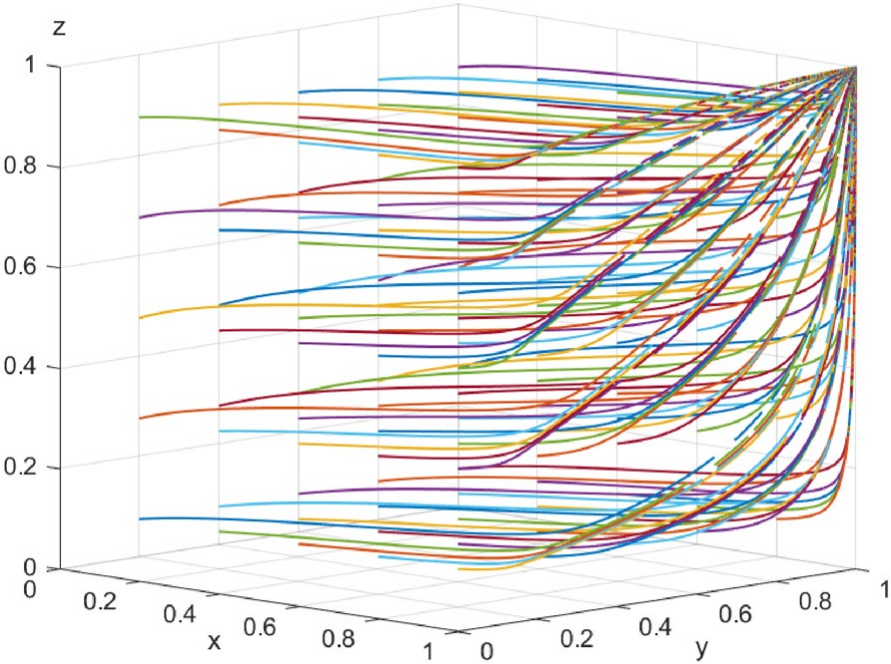
Simulation results of scenario 1 evolution.

**Figure 10:**
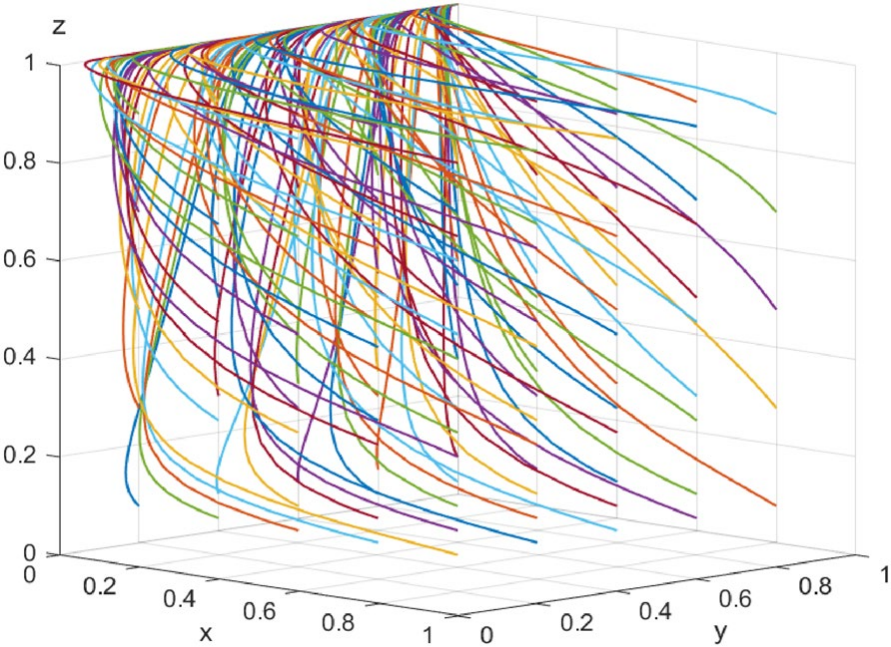
Simulation results of scenario 2 evolution.

**Figure 11:**
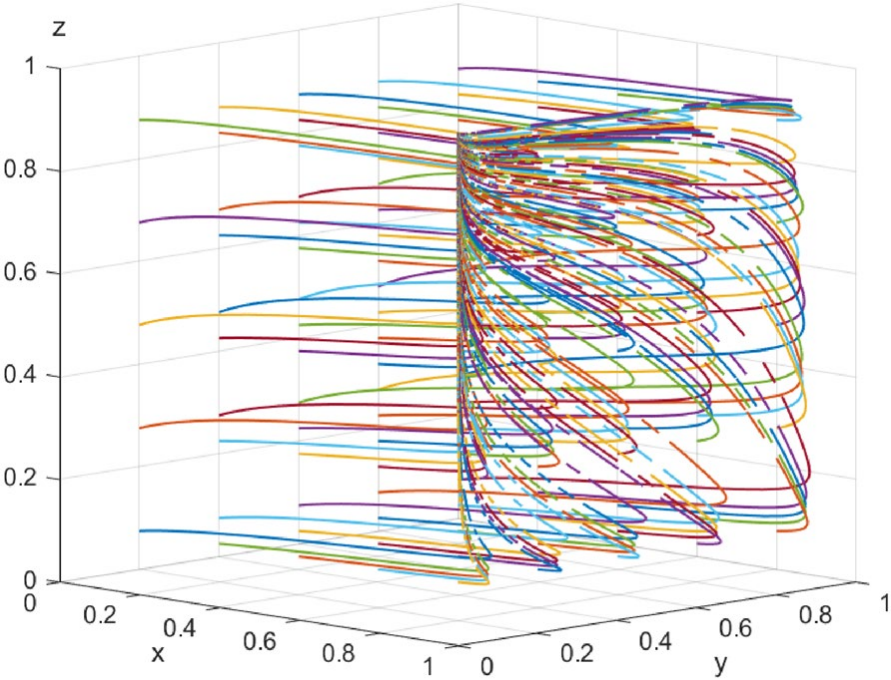
Simulation results of scenario 3 evolution.

**Figure 12:**
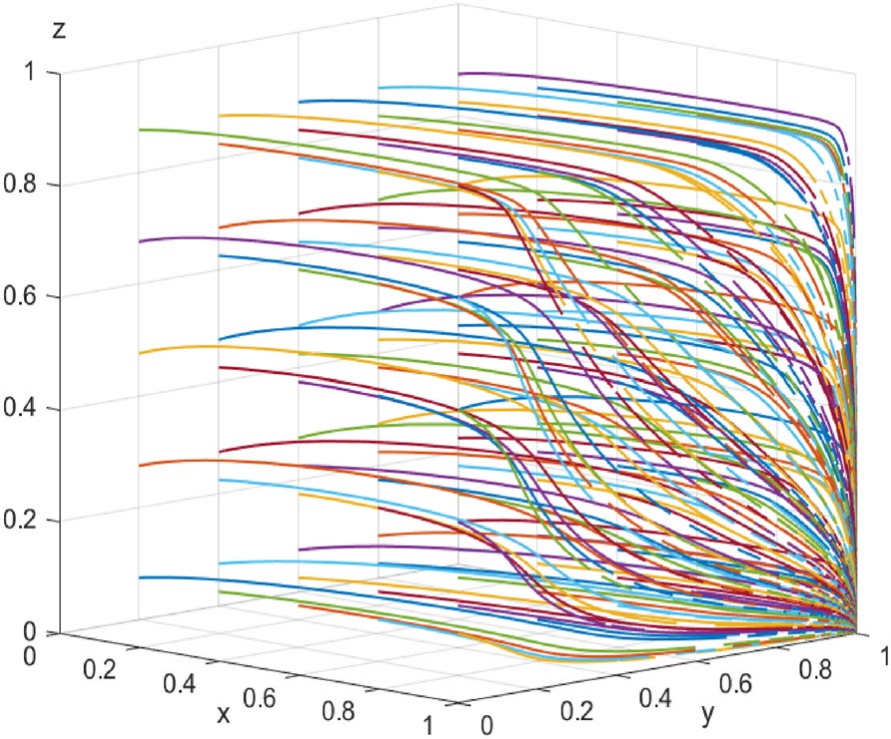
Simulation results of scenario 4 evolution.

**Figure 13:**
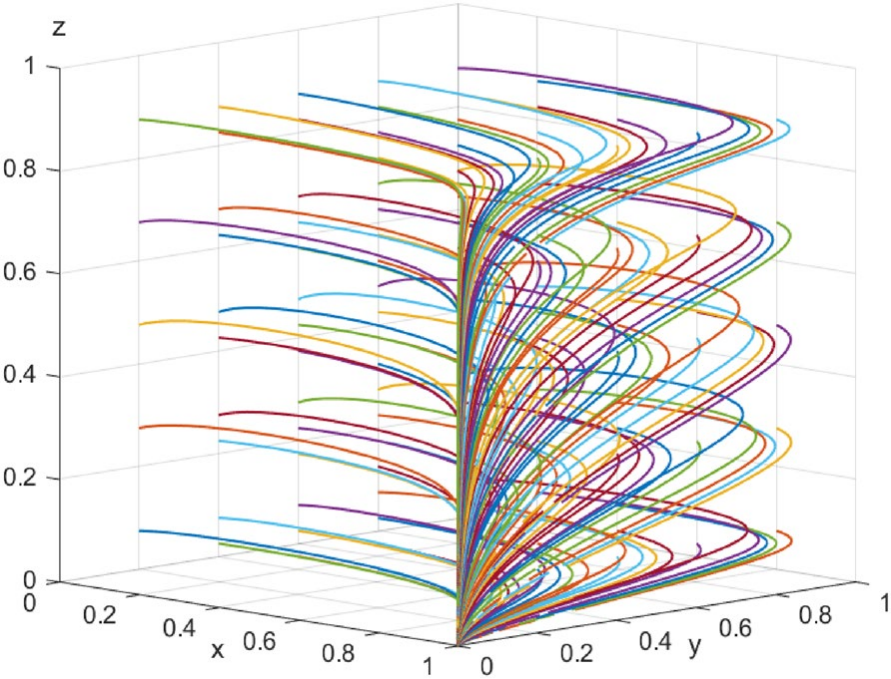
Simulation results of scenario 5 evolution.

**Figure 14:**
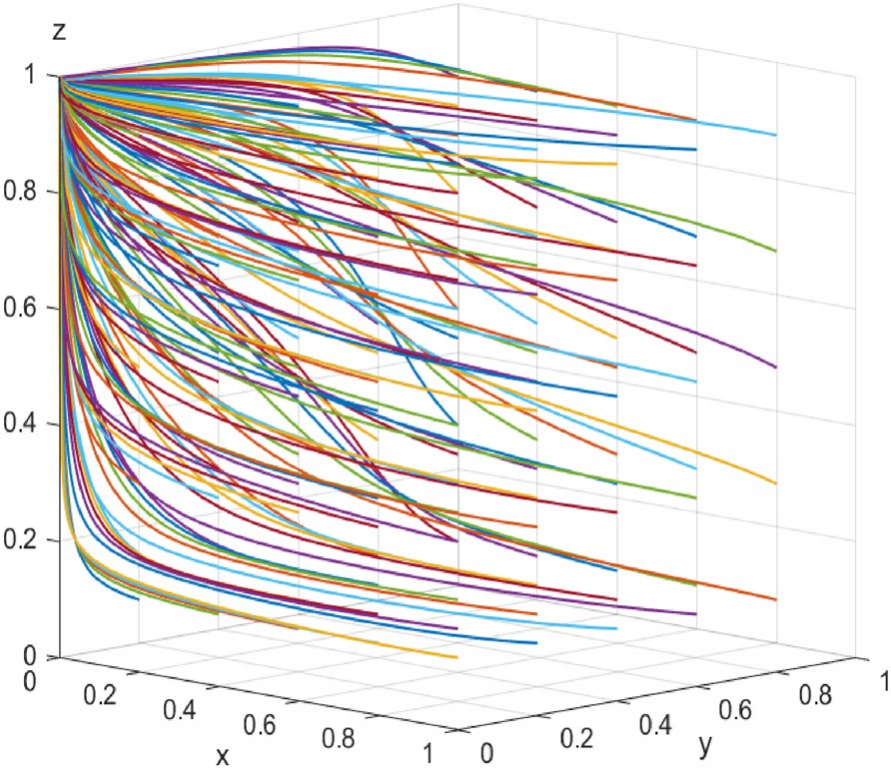
Simulation results of scenario 6 evolution.

The data simulation of the six evolutionary stabilization scenarios proposed in “Game model solving and analysis” is shown in Figs. 9, 10, 11, 12, 13 and 14: from the evolutionary results, it can be seen that array 1 satisfies the conditions of scenario 1, and at this time, there exists an evolutionary equilibrium point of the system $\left(1,1,1\right)$, the combination of the strategies of the enterprise-alliance, the government, and the end-user is (collaborative low-carbon distribution, reward and punishment combination policy, with low-carbon preference). By changing the value of ${\varphi }_{1}$ the value of the array 2 is adjusted to obtain array 2 satisfies the following case 2 conditions:${\mathcal{C}}_{x1}$=50*,* ${\mathcal{C}}_{x2}$=40, ${\mathcal{E}}_{1}=50$ *, *${\mathcal{E}}_{2}=35$*, *${\varphi }_{1}=255$*, *${\varphi }_{2}=250$*, *${\mathcalligra{m}}=0.5$*, *$S=5$*, *$A=1$*, *${\mathcal{C}}_{y1}=15$*, *${\mathcal{C}}_{y2}=20$*, *$\alpha =0.1$*, *$\mathcal{W}=10$*, *$\gamma =0.7$*, *$L=10$*, *${D}_{1}=4$*, *${D}_{2}=1$*, *$\beta =0.5$*, *${\mathcal{V}}=6$*,* $\delta =1$ . According to the simulation results Fig. 9, the evolutionary equilibrium point of the system at this time becomes $\left(0,1,1\right)$, and the strategy combinations of enterprise-alliance, government and end-users are (collaborative low-cost distribution, reward and punishment combination policy, with low-carbon preference). Therefore, when the revenue difference between collaborative low-cost distribution and collaborative low-carbon distribution of enterprise-alliance is relatively small, it will tend to choose collaborative low-carbon distribution, on the contrary, if the revenue difference is large, enterprise-alliance will tend to choose collaborative low-cost distribution. This result suggests that enterprise-alliance are not only seeking to maximize economic benefits, but are also willing to take part of the social responsibility of reducing carbon emissions. However, when the difference between the two revenues is large, the enterprise-alliance will still choose collaborative low-cost distribution by focusing on economic benefits. The simulation analysis is consistent with the conclusions of the stability analysis of each party's strategy and has the validity, which is of practical significance in guiding the choice of distribution strategy for enterprise alliances (other cases will not be verified for reasons of space).

### The impact of supply-side government policies on enterprise-alliance distribution strategies

**Figure 15:**
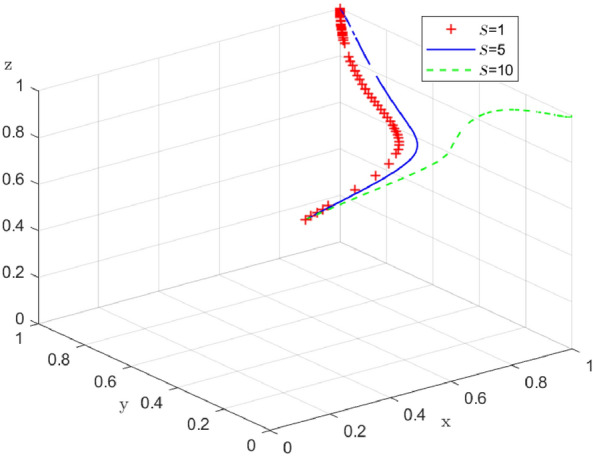
Impact of government reward on system evolution.

Since the combination of strategies in Case 1 (collaborative low-carbon distribution, reward and punishment combination policy, with low-carbon preference) is a desirable state of social operation, based on Case 1, the impact of government reward on enterprise-alliance distribution strategies is further discussed. When $S=(1,5,10),$ the results of 100 simulations of the replicable dynamic equation system evolving over time are shown in Fig. 15: in the process of the system evolving to a stable point, an increase in the government reward can accelerate the evolution of the enterprise-alliance to carry out collaborative low-carbon distribution. Enterprise-alliance will engage in collaborative low-carbon distribution in order to maintain their reputation with the government and end-users because of the rewards they receive from the government. The stronger the incentives from the government, the more stable the strategy of the enterprise-alliance in choosing collaborative low-carbon distribution. But with an increase in the government's financial burden, the government's financial burden increases, so the evolution of the government's policy of combining reward and punishment slows down when $S=10$ the financial burden is too heavy, and the government will turn to flow in the form of policy by reducing the cost of regulation or reducing incentives.

**Figure 16:**
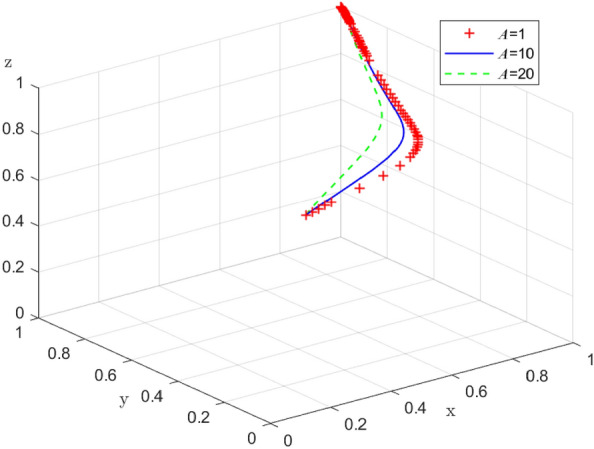
Impact of government punishment on system evolution.

Similarly, when enterprise-alliance collaborate on low-cost distribution, and government punishment $A=(1,10,20)$, the simulation results of replicating the system of dynamic equations evolving over time 100 times are shown in Fig. 16. During the evolution of the system to the stable point, an increase of government punishment can accelerate the evolution of the enterprise-alliance to carry out collaborative low-carbon distribution, and the enterprise-alliance will continue choosing collaborative low-carbon distribution in this case as long as the government's punishment for collaborative low-cost distribution is $A>0$.When the government punishment collaborative low-cost distribution, the enterprise-alliance will always choose collaborative low-carbon distribution in this case. This suggests that government punishment have a greater impact on enterprise-alliance's strategy choices than reward, because government punishment affect enterprise-alliance 's reputation in the industry as well as its credit rating.

### The impact of demand-side end-users’ behavior on the distribution strategies of enterprise-alliance

**Figure 17:**
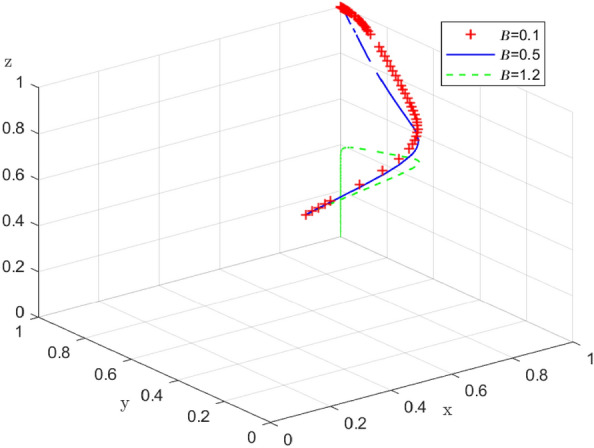
Impact of end-users’ low-carbon preference on system evolution.

Additionally, based on Case 1, we analyze the impact of end-users behavior on the distribution strategy of the enterprise-alliance, and first explore the impact of end-users with low-carbon preference who are willing to pay the low-carbon distribution subsidy coefficient $\beta$.The influence of the value of the subsidy coefficient of low-carbon preference is investigated $\beta =0.5$ .On the basis of $\beta =0.1$ and $\beta =1.2$ , the simulation results of 100 repetitions of the dynamic equation system evolving over time are shown in Fig. 17. According to the evolution results, when $\beta =0.1\text{and}0.5$, the end-users will tend to choose the low-carbon preference; when the subsidy coefficient $\beta$ is lower, the end-users with low-carbon preference consider it acceptable to spend extra distribution costs. However, when $\beta =1.2$, end-users no longer support the enterprise alliance in low-carbon distribution from the perspective of maximizing their own interests because the low-carbon distribution subsidy they have to pay is too high. It follows that the behavior of end-users is influenced by the costs they need to bear, and above a certain threshold they will no longer support enterprise-alliance for collaborative low-carbon distribution.

**Figure 18:**
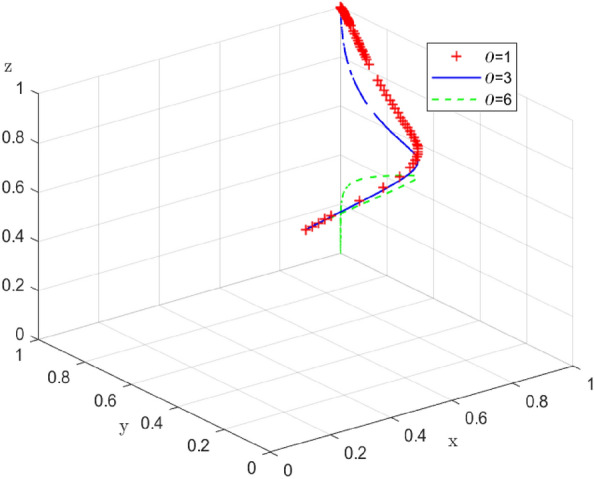
Impact of time cost on system evolution

Similarly, the simulation results of replicating the dynamic system of equations with time evolution 100 times when the time cost δ = (1, 3,6) that the end-users need to pay to support low-carbon distribution are shown in Fig. 18. From the evolution results, it can be seen that, when δ = 1 and 3, the end-users will tend to choose to support the enterprise-alliance in carrying out low-carbon distribution; But when δ = 6, the end-users will not support the enterprise-alliance in carrying out low-carbon distribution due to the high time cost that they need to pay. The current end-user requirements for the speed of logistics and distribution are increasingly high, if the enterprise-alliance in the collaboration of low-carbon distribution at the same time, logistics efficiency has not been improved, it will lead to the end-user's low-carbon preference is reduced until there is none.

## Conclusions and implications

This study aims to explore the dual effects of government reward and punishment mechanisms and end-users low-carbon preference on the choice of distribution strategies of enterprise-alliance. Through the government-led digitalization platform, the accurate accounting of cost data and carbon data of each distribution enterprise is realized, and the carbon trading market is effectively docked. Loss-of-benefit and carbon emission quantification models of collaborative low-carbon distribution modes and collaborative low-cost distribution modes are established, a dual incentive strategy considering government reward and punishment and end-user subsidies is proposed, and the effectiveness and feasibility of the models and strategies are verified through numerical experiments The main conclusions of this paper are as follows.

1. From the viewpoint of supply-side policy, the government should macroscopically control the reward and punishment mechanism according to the actual situation of the market^47^. Blindly increasing the reward will impose a heavy financial burden to the government, and with the development of the carbon trading market and increase in the carbon price, the government will gradually withdraw from the reward mechanism and play a major role in punishment. Compared with reward, strict punishment have a more obvious effect on the regulation of enterprise-alliance, so the government should consider local actuality, adopt a policy combination of dynamic reward and static punishment, further improve the construction of the carbon market, guide enterprises to actively participate in carbon trading, and give full play to the role of the carbon market.
2. Regarding demand-side end-users’ behavior, the end-user's support for the low-carbon distribution of the enterprise-alliance has a threshold, and after exceeding the threshold, the end-users will no longer support the enterprise-alliance in carrying out low-carbon distribution from the perspective of maximizing self-interest, so the enterprise-alliance should reasonably formulate the cost of low-carbon distribution, and guide the end-users to participate in the construction of the carbon market^48^.

The research presented in this paper further improves standard emissions and designs reasonable reward and punishment mechanisms^49^. However, the choice of enterprise-alliance strategies in real situations is often influenced by other constraints, while the evolutionary game model in this study is based on assumptions and the choice of parameters is idealized. Future research directions should combine the actual situation of distribution development and comprehensively consider the multiple factors involved in enterprise-alliance distribution.

## Data Availability

The datasets used and analysed during the current study available from the corresponding author on reasonable request.
